# Approaches to risk–benefit assessment of seafood consumption: lessons learned from an evidence scan

**DOI:** 10.3389/fnut.2024.1290680

**Published:** 2024-02-15

**Authors:** Sofia M. Santillana Farakos, Jacqueline Heilman, Eileen Abt, Sherri Dennis

**Affiliations:** Center for Food Safety and Applied Nutrition, United States Food and Drug Administration, College Park, MD, United States

**Keywords:** risk–benefit, risk analysis, RBA, fish, risk management

## Abstract

Qualitative and quantitative risk–benefit assessments (RBA) can be used to support public health decisions in food safety. We conducted an evidence scan to understand the state of the science regarding RBA in seafood to help inform seafood dietary advice in the United States. We collected published RBA studies assessing seafood consumption, designed inclusion and exclusion criteria to screen these studies, and conducted systematic data extraction for the relevant studies published since 2019. Our findings indicate the selection of health risks and benefits does not generally follow a systematic approach. Uncertainty and variability in RBAs is often not addressed, and quantitative RBAs making use of a single health metric generally have not been leveraged to directly support published regulatory decisions or dietary guidance. To elevate the role of RBA in supporting regulatory decision-making, risk assessors and risk managers must work together to set expectations and goals. We identified the need for a prioritization phase (e.g., multicriteria decision analysis model) to determine the risks and benefits of greatest public health impact to inform the RBA design. This prioritization would consider not only the degree of public health impact of each risk and benefit, but also the potential for risks and benefits to converge on common health outcomes and their importance to subpopulations. Including a prioritization could improve the utility of the RBAs to better inform risk management decisions and advance public health. Our work serves to guide the United States Food and Drug Administration’s approaches to RBA in foods.

## 1 Introduction

Seafood can be a source of exposure to contaminants [e.g., methylmercury (MeHg), dioxins and dioxin-like polychlorinated biphenyls (PCBs), microbial pathogens] as well as nutrients [e.g., n-3 polyunsaturated fatty acids (PUFAs), vitamin D, vitamin B_12_, iodine and selenium] (1). Although contaminants in seafood have been associated with health risks, the failure to incorporate beneficial nutrients and other factors provided by seafood in the diet can likewise be considered a health risk.

Risk–benefit assessments (RBAs) for foods emerged to support the integration of health-relevant evidence from independent nutritional and risk-related assessments (2). The first RBA for food was published in 1999 by Chan et al. (3) on fish consumption in the Kahnawake community (4). RBAs independently estimate risks and benefits resulting from exposure and then integrate and compare them. The probability of adverse health effects (risk) is weighed against the probability of beneficial health effects (5). RBAs can be qualitative, semiquantitative or quantitative. A qualitative RBA is dependent on expert elicitation, while a semiquantitative RBA is data-driven and uses qualitative information to inform a measured approach. A quantitative RBA integrates risks and benefits using a single health metric [e.g., Disability Adjusted Life Years (DALY)].

The field of RBA for foods is still emerging (2). A systematic review of RBA conducted by Boué et al. (4) identified 47 RBAs for fish published prior to 2014 including the 2009 draft quantitative assessment published by the United States (US) Food and Drug Administration (FDA) on the net effects on fetal neurodevelopment from consumption of commercial fish, finalized in 2014 (6). A recent scoping review by Thomsen et al. (1) from 2000–2019 identified 106 published RBAs of fish and other seafood (1), indicating an increase in the number of published RBAs (4).

The aim of our study was to conduct an evidence scan of the state of the science regarding RBA in seafood to inform the process of seafood dietary advice development in the United States. An evidence scan follows a similar methodology to that of a systematic review with a rigorous approach and an analytical framework. However, while a systematic review addresses a formulated question and identifies, selects, analyzes, and synthesizes evidence, an evidence scan involves a thorough search of the literature to gather new and relevant evidence on a topic and does not include evaluation of the data (7). The National Academies of Science, Engineering, and Medicine (NASEM) has convened a committee jointly sponsored by the Department of Health and Human Services, the Environmental Protection Agency, the Department of Agriculture, and the Department of Commerce to review the role of seafood consumption in child growth and development (8). As part of their review, the committee is charged with evaluating when to (or not to) conduct a risk benefit analysis. The results of our evidence scan provide a basis for FDA discussions of RBA for foods and contributed to informing the charge questions for the NASEM Committee on the Role of Seafood in Child Growth and Development.

## 2 Materials and methods

### 2.1 Search terms

We identified published RBA studies related to seafood consumption through literature searches in PubMed, CINAHL, Cochrane Central Register of Controlled Trials (CENTRAL), Embase and Google Scholar, Search terms are provided in the Supplementary Material S1.

### 2.2 Search dates

Thomsen et al. (1) conducted a scoping review of seafood RBAs for studies dated from 2000 through April 2019. As such we restricted our search to publications from April 2019 forward. Our search was completed August 1, 2023, and returned 1,635 citations. An additional 115 studies were added to our captured citations, and these 115 consisted of 106 identified by Thomsen et al. (1) plus additional studies identified by Boué (4) (duplicates removed).

### 2.3 Inclusion/exclusion criteria, protocol, and screening

Inclusion and exclusion criteria were used to screen for identified abstracts and capture seafood RBAs (Table 1). Abstracts were selected for full text review if publications were in English, and if study designs considered seafood consumed as a whole (not as a component of a specific diet or as fish extracts or supplements). RBAs retained for review were restricted to those that pertained to public health and excluded disciplines other than food and nutrition, such as economics, sociology, animal welfare, and the environment. Only studies considering risks and benefits were retained. Allergy as a risk was excluded because of the specificity of the affected population. Two independent reviewers screened abstracts for inclusion/exclusion. Two additional independent arbiters served as tiebreakers when the independent reviewers’ conclusions disagreed. Abstracts were screened using Covidence (9).

**Table 1:** Inclusion and exclusion criteria for abstract screening used in the RBA evidence scan.

Criteria	Inclusion	Exclusion
Study design	Qualitative RBA Quantitative RBA Semiquantitative RBA Position papers on risks and benefits Methodology reviews/critiques Reviews of RBA Meta-analysis of RBA	Conference Abstracts Unpublished studies Irretrievable studies Publications unavailable in English Intervention of fish extracts or supplements Full diet studies with fish component
Scope	Nutrition (food sector) Toxicology Neurodevelopment/development	Economy or cost Sociology Animal welfare Environmental impact or sustainability Studies specific to any fields other than food/nutrition
Title	An RBA A study of RBA introducing a comparison of risk and benefit Methodology or critique of RBA	No criteria
Research aims	Comparison of the risks and benefits of food Review of the beneficial and adverse health effects due to consumption of a specific food (fish/seafood)	No criteria
Outcomes	Disease indicators Health benefits Composite matrix such as DALY/QALY Food safety thresholds Risk Net risk or net benefit	Allergy Only risks or only benefits, not both
Population/Location	Low, medium, high or very high human development Preferably North America	No criteria

### 2.4 Screening for full text review and data extraction

We designed a systematic data extraction protocol for relevant studies identified through abstract screening. Our objective was to gather information regarding RBA methodologies, including study design and performance, and whether study conclusions were leveraged to support dietary recommendations. We extracted the following elements from each study: methodology, main results, investigators’ institutional affiliation, country, year, population, research question(s), food type and granularity level (e.g., species of fish, cut of fish, and cooking methods as available), food intake estimate methods, substances associated with risks (risk agents), adverse health effects associated with the risk agents, substances associated with benefits (benefit agents), beneficial health effects associated with benefit agents, methods for assessing exposure to the risk and benefit (e.g., measurement of nutrients and chemicals in fish tissue), rationale for selected risks and benefits, RBA type (qualitative, semiquantitative, quantitative) as defined by tier level according to European Food Safety Authority (5) (EFSA) guidelines, identity of semi-quantitative or single health metrics if used, declaration of a link with published guidance or policy, rationale for study methodology, author-conducted quality assessment, consideration of variability and uncertainty, and any additional pertinent information.

We extracted data from all relevant studies dated 2019 or later. Extracted data were collected in Microsoft Excel (2018).

## 3 Results

### 3.1 Number of studies, year, country of origin, and population of interest

A total of 1,750 publication abstracts were screened. 227 were selected for full text review, of which 116 were published since 2019. Upon full text review, 33 studies did not undergo data extraction as they were either abstracts for conferences, duplicates, erratum to other publications, updates to previously published RBAs, or not RBAs. We extracted data from 83 RBA studies (references listed in alphabetical order in Supplementary Material S1).

Of the studies that underwent data extraction, 17% were published in 2019, 19% in 2020, 16% in 2021, 31% in 2022 and 17% in 2023 (January through August 1st). Various populations, fish sources, and consumption data were represented, including a majority from Europe, followed by Asia, North America, Africa, and then other locations. Most studies (67%) investigated the general population (or additional details were not provided), 11% investigated adults, 6% children, 12% explicitly children and adults and 3% included pregnant woman.

### 3.2 Food and intake estimates

Forty-seven percent of studies investigated marine fish, 17% freshwater fish, 17% shellfish, 7% a mixture of marine fish and shellfish, 5% a mixture of marine and freshwater fish, 2% mixture of freshwater fish and shellfish, and 5% a combination of marine fish, freshwater fish, and shellfish. Approximately 86% percent of the studies reported fish being fresh (uncooked), 4% processed and 10% included a mix of fresh and processed. Further details on seafood type were not consistently reported. While some studies reported information to the species level others reported food simply as fish, seafood, or shellfish. In addition, some studies reported the collection locations, biometrics of fish, cooking details and cuts of fish (e.g., dorsal, ventral, organs).

Intake estimate methods varied among studies and were not consistently reported. Intake estimates either relied on assumptions (e.g., 6-ounce portion size for an adult, twice per week), market information (e.g., sales of particular types of fish), survey data, and government data or literature. Other studies did not report intake estimates or the RBA comparison relied on characteristics of the fish studied (e.g., MeHg content) and thus did not require intake estimates.

### 3.3 RBA type, selection of risks and benefits methodology

Of the extracted studies, 18% were qualitative, 16% quantitative [one study being on food substitution (10)] and the majority (66%) were semiquantitative. Single health metrics from the quantitative RBAs included intellectual quotient (IQ), visual recall memory (VRM) or other learning/memory metrics, probability of cardiovascular outcomes, and DALY. For semiquantitative studies, health benefit value (HBV) for selenium (Se) or other hazard indices or quotients were used.

**Figure 1:**
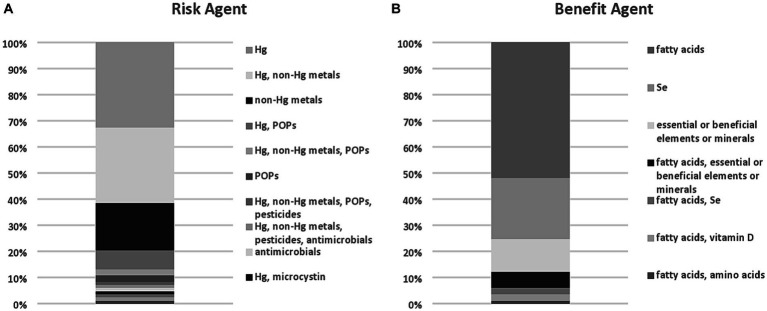
Agents of risk **(A)** and benefit **(B)** in relevant RBA published studies identified through the evidence scan.

Mercury was the most frequently assessed risk agent, while fatty acids were the most frequently assessed benefit agent (Figure 1). Other risk agents included persistent organic pollutants (POPs) such as dioxins, PCBs, and brominated flame retardants, toxic elements or other metals (e.g., nickel, tin) and pesticides. Two RBAs included antimicrobials (11, 12), one investigated microcystins (13), and one included PFAS as a risk agent (14). An additional study by Marquès et al. (15) included PFAS as a risk agent and was identified in the search but was not considered for our analysis as it was an update to an RBA conducted prior to 2019. Other benefit agents included Se, essential or beneficial elements, or minerals (including vitamin D and amino acids). The rationale for the choice of risks and benefits was typically not described by investigators. Of those studies that did provide some rationale, several based the selection on the role of Se as an antagonist to reactive oxygen species, others included as many identifiable risks and benefits as possible, and a few selected the risks and benefits based on previous studies or expert judgment. In many cases, it appeared that the risks or benefits selected were motivated by the investigators’ analytical capabilities. Quantitation of risk or benefit agents was most often conducted *de novo* in the seafood assessed by the investigators (~90% of studies), but in some cases, RBAs leveraged published literature or databases (~10%) to estimate levels of risk or benefit agents in the seafood assessed.

### 3.4 Quality assessment, handling of variability and uncertainty, link with policy

A few studies conducted quality assessments by comparing their analytical results with other published values (e.g., reproducibility) (16–27). Variability and uncertainty analyses were generally not included in the published studies, although a few addressed this as part of their discussion on limitations. A few studies used probabilistic methods to support the RBA and included uncertainty analysis (28–30). None of the RBA studies mentioned being commissioned to inform dietary guidance or regulatory decisions on seafood consumption. A review by Lemming and Pitsi (31) presenting information on food consumption and nutrient intake in the Nordic and Baltic countries to inform the 2022 update of the Nordic Nutrition recommendations did not meet our inclusion criteria. However, the Norwegian Scientific Committee for Food and Environment (VKM) subsequently published a benefit and risk assessment of fish in the Norwegian diet, which included development of a quantitative benefit assessment and independent semiquantitative benefit and risk assessments (32). The quantitative assessment was limited to benefits (did not include risks) because the extensive literature review conducted did not reveal adverse health outcomes to be associated with fish intake.

## 4 Discussion

Previous reviews of seafood RBAs include those published by Thomsen et al. (1) and Boué et al. (4). Like the findings of Thomsen et al. (1) that addressed RBAs published before 2019, we also found that most studies published since 2019 were semiquantitative, with exposure being compared to an established health guidance value (e.g., RfD, TDI, DRV, HBGVs). While Thomsen et al. (1) identified the QALY as the primary health metric used, we found it was the DALY. We did not find DALY-or QALY-based optimization studies, but we did identify the study by Thomsen et al. (10) that used DALYs to assess substitution of red meat for fish. We also found that studies typically address a specific country or region and their general population, with recommendations that further analysis be done for potentially susceptible subpopulations.

Only a small percentage of studies provided the rationale behind their selection of risks and benefits. Studies tended to select risks and benefits without applying a strict systematic approach. Other studies took an inclusive approach, where every accessible risk or benefit was considered. In many cases the selection of risks and benefits appeared driven by the analytical capabilities of the investigators or the nature of the available data. For example, in some cases, investigators used locally available contaminant data with international health guidance values for comparison; possibly because the ideal data was beyond reach or scope. Moreover, as reported by Thomsen et al. (1), the methodology applied for RBAs tended to be linked to the selected benefits and risks included in the analysis. This has also been identified as a challenge by Pires et al. (33). In part to try to address this issue, Boué et al. (34) proposed a harmonized methodological framework for selection of nutritional, microbiological, and toxicological RBA components. Additionally, EFSA is planning an update of its 2010 Guidance on the Human Health Risk–Benefit Assessment of Foods which may inform approaches to the selection of risks and benefits (35).

We found variability and uncertainty analysis is typically not conducted for RBAs, and validation of the RBA method is not regularly conducted or discussed. Similarly, Pires et al. (33) and Thomsen et al. (1) identified uncertainty and variability as key challenges in RBA. Uncertainty in RBA derives from the methodology applied in the RBA as well as from the sources of uncertainty that would be present in the corresponding individual risk assessment(s) and benefit assessment(s). Although EFSA and BRAFO recommend that at a minimum qualitative assessment of uncertainties be conducted (5, 36), an assessment or even discussion of uncertainty in the RBA and a validation of the RBA have generally not been conducted.

We generally did not find published evidence of RBA conclusions being leveraged to inform policy advice from authoritative bodies, except for a benefit and risk assessment of fish published by VKM (32). This could indicate a potential misalignment between the utility of RBA conclusions derived by risk assessors and the needs of risk managers. It could also suggest that as uncertainty and complexity of RBAs increase, risk managers may be less inclined to leverage RBAs in risk management decisions and communications.

The application of RBA by risk managers in establishing dietary recommendations will require RBAs to be increasingly practical, comprehensible, and easily communicated. Accomplishing that for seafood is challenging as seafood, possibly more than any other food type, comprises many risks and benefits. Some of the risk agents and benefit agents are mechanistically related and impact a common health outcome. For example, Se has a direct role in maintaining functional selenoenzymes and this provides protection against MeHg induced neurotoxicity (37). There are also mechanistically unrelated risk agents and benefit agents in seafood that do not directly counteract each other, but ultimately modulate a common health outcome, such as mercury and PUFA modulation of cardiovascular risk. Finally, seafood is a source of unrelated, individual risk agents such as PCBs or PFAS, and unrelated, individual benefit agents such as Vitamin D or iodine. Each of these three categories of risks and benefits can be assessed separately using semi-quantitative RBAs, and indeed we found semi-quantitative RBA is the most frequently conducted type of seafood RBA. However, a single semi-quantitative RBA may not be capable of considering all important risks and benefits concurrently and arriving at a comprehensive and useful conclusion to convey to risk managers. A single health metric (DALY or QALY) must be used instead. But for this, a quantitative understanding of disease burden is required and this information may not be available for the complete set of risks and benefits and may only be in development for risk agents such as PFAS mixtures (38).

Rather than conducting a semi-quantitative RBA for a single risk agent and a single benefit agent, a fully quantitative RBA based on a single health metric, or an RBA based on a final ranked list of components (34), a prioritization step could first be established to determine the risks and benefits with the greatest public health impact [e.g., using a multicriteria decision analysis model (MCDA)]. The MCDA can be established within the context of risk managers’ values, goals, and understanding of outcomes, which will vary by region, population, consumption patterns and other factors. This prioritization would serve as a first step in a stepwise approach to RBA. From each individual risk or benefit, or each risk–benefit pair (whether mechanistically related or not), the risk–benefit combination with the greatest potential public health impact could be selected as the first qualitative or semi-quantitative RBA to be conducted. If that qualitative or single semi-quantitative RBA does not meet the needs of risk managers or match the competing priorities of the scenarios (multiple hazards and multiple benefits), assessors can conduct a subsequent semi-quantitative RBA, informed by the prioritization, that further refines the results of the first RBA. The proposed systematic prioritization would help focus the analysis on the risks and benefits of greatest public health impact, and the use of successive RBAs could allow for a flexibility where additional or emerging risks, benefits, and public health priorities could be incorporated as necessary to make the process fit for purpose. The advantage of this prioritization step is reflected in the quantitative benefit and independent benefit and risk assessments published by VKM (32) where initial findings demonstrated no strong evidence of an adverse effect from fish consumption based on health outcomes in children or adults, and thus the quantitative assessment focused solely on benefits. Because this approach entails values-based decisions and refinements such as the selection of priority subpopulations, economic or environmental impacts, and feasibility, it is important for risk managers to be engaged from the onset. This prioritization scheme would be applicable to seafood as well as any other food commodity.

In conclusion, our analysis shows that the linkage between RBA, risk–benefit management decisions, and dietary recommendations communicated to the public needs to be strengthened. Our proposed incorporation of a prioritization phase in the RBA, guided by the needs and values of policy-and decision-makers, together with successive refining of the RBA, could improve the utility of RBA to inform risk management decisions and advance public health.

## Author contributions

SS: Conceptualization, Data curation, Formal analysis, Investigation, Methodology, Project administration, Visualization, Writing – original draft, Writing – review & editing. JH: Conceptualization, Data curation, Formal analysis, Investigation, Methodology, Visualization, Writing – original draft, Writing – review & editing. EA: Validation, Writing – review & editing. SD: Conceptualization, Project administration, Supervision, Validation, Writing – review & editing.
