# Supplementary material for: Approaches to risk–benefit assessment of seafood consumption: lessons learned from an evidence scan
Source: Front Nutr. 2024 Feb 15;11:1290680. doi: 10.3389/fnut.2024.1290680 (PMC10902146; doi:10.3389/fnut.2024.1290680)
Supplement: Supplementary file 1 [file Data_Sheet_1.docx]

Supplementary Material

*Approaches to risk-benefit assessment of seafood consumption: lessons learned from an evidence scan*

Sofia M. Santillana Farakos^ⴕ^*, Jacqueline Heilman^ⴕ^, Eileen Abt, and Sherri Dennis

*Corresponding author sofia.santillanafarakos@fda.hhs.gov

1. **List of search terms for RBA and Seafood**

- RBA “disease burden” OR “risk benefit” OR “risk-benefit” OR “risk/benefit” OR “benefit-risk” OR "disability adjusted life year" OR DALY[tiab] OR "quality adjusted life year" OR QALY[tiab] OR (Risk* AND Benefit*)
- Seafood: Seafood OR "Seafood"[Mesh] OR seafoods OR sea-food OR “sea food” OR sea-foods OR fish OR “fish consumption” OR "Fishes"[Mesh] OR fishes OR "Fish Proteins"[Mesh] OR “fish proteins” OR “fish products” OR “fish flour” OR “fatty fish” OR shellfish OR “shellfish proteins” OR “mercury poisoning” OR "Mercury Poisoning"[Mesh] OR methylmercury OR "Sharks"[Mesh] OR sharks OR swordfish OR "Tuna"[Mesh] OR tuna OR "Salmon"[Mesh] OR salmon OR sardines OR "Gadiformes"[Mesh] OR pollock OR "Flounder"[Mesh] OR flounder OR cod OR "Tilapia"[Mesh] OR tilapia OR shrimp OR "Ostreidae"[Mesh] OR oysters OR "Mya"[Mesh] OR "Bivalvia"[Mesh] OR clams OR "Pectinidae"[Mesh] OR scallops OR "Brachyura"[Mesh] OR crab OR "Perciformes"[Mesh] OR mackerel OR “Catfishes"[Mesh] OR catfishes OR "Trout"[Mesh] OR trout OR lobster OR "Decapodiformes"[Mesh] OR squid OR halibut OR “mahi mahi” OR crawfish OR anchov* OR herring OR rockfish OR marine product* OR “marlin” OR “orange roughy” OR “tilefish” OR “whales”[Mesh] OR “perches”[Mesh] OR walleye OR “bass”[Mesh] OR “lake trout OR salmonid OR catfish OR sushi OR ceviche OR sashimi OR gravlax OR lox OR “tuna tartare” OR “seafood crudo” OR “fluke crudo” OR fluke OR carpaccio OR “e'la ota” OR poke OR hinava OR “gohu ikan” OR hoe OR esqueixada OR kuai OR kelaguen OR namero OR kilawin OR stroganina OR yusheng OR “koi pla” OR Kokoda OR kuai OR lakerda OR “larb pla” OR “ota ika” OR tiradito OR xató OR umai OR “Salmo salar”[Mesh] OR salmon salar* OR Atlantic Salmon OR salmonids”[Mesh] OR Salmonids.

1. **List of relevant studies in alphabetical order by reference as identified in the evidence scan including the country and RBA type.**

| RBA Study Reference | Country | RBA Type |
| --- | --- | --- |
| Afonso, Bernardo (1) | Portugal | Semiquantitative |
| Alam, Sumaila (2) | Philippines | Semiquantitative |
| Annibaldi, Truzzi (3) | Italy | Semiquantitative |
| Barchiesi, Branciari (4) | Italy | Semiquantitative |
| Barone, Storelli (5) | Italy | Semiquantitative |
| Bernstein, Oken (6) | United States | Qualitative |
| Branciari, Franceschini (7) | Italy | Semiquantitative |
| Bridges, Furin (8) | United States | Semiquantitative |
| Cai, Zhu (9) | China | Quantitative |
| Carvalho, Correia (10) | Portugal | Quantitative |
| Chang, Chiang (11) | Taiwan | Semiquantitative |
| Cressey, Miles (12) | New Zealand | Quantitative |
| De Cock, Forio (13) | Ecuador | Semiquantitative |
| Dellinger, Anguzu (14) | USA | Semiquantitative |
| Fan, Zou (15) | China | Semiquantitative |
| Fang, Liang (16) | China | Semiquantitative |
| Farzad, Kuhn (17) | United States | Semiquantitative |
| Fujimura and Yoshinaga (18) | Japan | Quantitative |
| Girolametti, Annibaldi (19) | Italy | Qualitative |
| Gladyshev, Anishchenko (20) | Russia | Semiquantitative |
| Grgec, Kljakovic-Gaspic (21) | Croatia | Semiquantitative |
| Gui, Wang (22) | China | Semiquantitative |
| Gui, Wang (23) | China | Quantitative |
| Hasselberg, Nostbakken (24) | Ghana | Qualitative |
| Hollander, De Jonge (25) | The Netherlands | Quantitative |
| Irigoyen-Arredondo, Moreno-Sánchez (26) | Mexico | Qualitative |
| Jing, Lin (27) | China | Quantitative |
| Kendler, Thornes (28) | Norway | Qualitative |
| Kosker (29) | Turkey | Semiquantitative |
| Lara, Galván-Magaña (30) | Mexico | Semiquantitative |
| Lazarini, Milani (31) | Brazil | Semiquantitative |
| Lin, Nan (32) | Taiwan | Semiquantitative |
| Lin, Lee (33) | Taiwan | Quantitative |
| Łuczyńska, Nowosad (34) | Poland | Semiquantitative |
| Łuczyńska, Łuczyński (35) | Poland | Semiquantitative |
| Maia, Almeida (36) | Portugal | Semiquantitative |
| Medina-Morales, Corro-Espinosa (37) | Mexico | Semiquantitative |
| Melgar, Núñez (38) | Spain | Semiquantitative |
| Meng, Wang (39) | China | Semiquantitative |
| Mesa, Gil (40) | Spain | Qualitative |
| Milićević, Romanić (41) | Croatia | Semiquantitative |
| Mititelu, Neacșu (42) | Romania | Semiquantitative |
| Noger-Huet, Vagner (43) | France | Semiquantitative |
| Nøstbakken, Rasinger (44) | Norway | Qualitative |
| Okati, Shahriari Moghadam (45) | Iran | Semiquantitative |
| Özden, Erkan (46) | Turkey | Qualitative |
| Öztürk (47) | Turkey | Semiquantitative |
| Peycheva, Panayotova (48) | Bulgaria | Semiquantitative |
| Peycheva, Panayotova (49) | Bulgaria | Semiquantitative |
| Peycheva, Panayotova (50) | Bulgaria | Semiquantitative |
| Plessl, Gilbert (51) | South Africa | Semiquantitative |
| Prato, Biandolino (52) | Italy | Qualitative |
| Harb Rabia, Luzardo (53) | Spain | Semiquantitative |
| Rajkowska-Myśliwiec, Pokorska-Niewiada (54) | Poland | Qualitative |
| Ralston, Kaneko (55) | United States | Semiquantitative |
| Ricketts, Voutchkov (56) | Jamaica | Semiquantitative |
| Roila, Branciari (57) | Italy | Semiquantitative |
| Sabino, Bodin (58) | Seychelles | Semiquantitative |
| Sardenne, Bodin (59) | Seychelles | Semiquantitative |
| Sepúlveda, Sotelo-Gonzalez (60) | Mexico | Semiquantitative |
| Simukoko, Mwakalapa (61) | Zambia | Semiquantitative |
| Sofoulaki, Kalantzi (62) | Greece | Semiquantitative |
| Song, Fang (63) | China | Semiquantitative |
| Strandberg, Bhavsar (64) | Canada | Qualitative |
| Sun, Wang (65) | China | Qualitative |
| Suyani, Rajesh (66) | India | Semiquantitative |
| Thomsen, de Boer (67) | Denmark | Quantitative |
| Tuomisto, Asikainen (68) | Denmark, Estonia, Finland, Sweden | Quantitative |
| Uçar (69) | Turkey | Semiquantitative |
| Ulusoy, Mol (70) | Turkey | Semiquantitative |
| Ulusoy and Mol (71) | Turkey | Semiquantitative |
| Varol, Kaçar (72) | Turkey | Qualitative |
| Varol, Kaçar (73) | Turkey | Qualitative |
| Varol and Kaçar (74) | Turkey | Qualitative |
| Wang, Dong (75) | China | Quantitative |
| Wang, Chen (76) | China | Quantitative |
| Xie, Liu (77) | China | Semiquantitative |
| Yabanli and Tay (78) | Turkey | Semiquantitative |
| Yánez-Jácome, Romero-Estévez (79) | Ecuador | Semiquantitative |
| Yin, Chen (80) | China | Quantitative |
| Zamora-Arellano, Betancourt-Lozano (81) | Mexico | Semiquantitative |
| Zihao, Qian (82) | China | Semiquantitative |
| Zou, Yin (83) | China | Semiquantitative |

1. Afonso C, Bernardo I, Bandarra NM, Martins LL, Cardoso C. The Implications of Following Dietary Advice Regarding Fish Consumption Frequency and Meal Size for the Benefit (Epa + Dha and Se) Versus Risk (Mehg) Assessment. *Int J Food Sci Nutr* (2019) 70(5):623-37. doi: 10.1080/09637486.2018.1551334.

2. Alam L, Sumaila UR, Bari MA, Rusydy I, Musthafa MS, Mokhtar M. Investigating Fish Contamination Scenario and Community Willingness to Adopt Consumption Advice Proposing an Advisory Option. *Environ Sci Pollut Res Int* (2022) 29(16):24167-79. Epub 20211125. doi: 10.1007/s11356-021-17715-9.

3. Annibaldi A, Truzzi C, Carnevali O, Pignalosa P, Api M, Scarponi G, et al. Determination of Hg in Farmed and Wild Atlantic Bluefin Tuna (Thunnus Thynnus L.) Muscle. *Molecules* (2019) 24(7):Article #: 1273. doi: 10.3390/molecules24071273.

4. Barchiesi F, Branciari R, Latini M, Roila R, Lediani G, Filippini G, et al. Heavy Metals Contamination in Shellfish: Benefit-Risk Evaluation in Central Italy. *Foods* (2020) 9(11). doi: 10.3390/foods9111720.

5. Barone G, Storelli A, Meleleo D, Dambrosio A, Garofalo R, Busco A, et al. Levels of Mercury, Methylmercury and Selenium in Fish: Insights into Children Food Safety. *Toxics* (2021) 9(2). doi: 10.3390/toxics9020039.

6. Bernstein AS, Oken E, De Ferranti S, Lowry JA, Ahdoot S, Baum CR, et al. Fish, Shellfish, and Children’s Health: An Assessment of Benefits, Risks, and Sustainability. *Pediatrics* (2019) 143(6). doi: 10.1542/peds.2019-0999.

7. Branciari R, Franceschini R, Roila R, Valiani A, Pecorelli I, Piersanti A, et al. Nutritional Value and Contaminant Risk Assessment of Some Commercially Important Fishes and Crawfish of Lake Trasimeno, Italy. *International Journal of Environmental Research and Public Health* (2020) 17(7):2545.

8. Bridges KN, Furin CG, Gerlach RF. Subsistence Fish Consumption in Rural Alaska: Using Regional Monitoring Data to Evaluate Risk and Bioavailability of Dietary Methylmercury. *Science of the Total Environment* (2020) 736. doi: 10.1016/j.scitotenv.2020.139676.

9. Cai H, Zhu Y, Qin L, Luo B, Liu H, Wu C, et al. Risk-Benefit Assessment of Methylmercury and N-3 Polyunsaturated Fatty Acids through Fish Intake by Pregnant Women in Shanghai, China: Findings from the Shanghai Diet and Health Survey. *Food and Chemical Toxicology* (2023) 174. doi: 10.1016/j.fct.2023.113668.

10. Carvalho C, Correia D, Severo M, Afonso C, Bandarra NM, Gonçalves S, et al. Quantitative Risk-Benefit Assessment of Portuguese Fish and Other Seafood Species Consumption Scenarios. *British Journal of Nutrition* (2022) 128(10):1997-2010. doi: 10.1017/S0007114521004773.

11. Chang CH, Chiang CF, Liao JW, Yen GC, Huang YC, Ni SP, et al. Dietary Exposure Assessment of Methylmercury and Polyunsaturated Fatty Acids in Saltwater Fish and Processed Foods among Taiwanese Women of Child-Bearing Age and Children: A Novel Core Food-Matching Approach. *Chemosphere* (2021) 262. doi: 10.1016/j.chemosphere.2020.128249.

12. Cressey P, Miles G, Saunders D, Pearson AJ. Mercury, Methylmercury and Long-Chain Polyunsaturated Fatty Acids in Selected Fish Species and Comparison of Approaches to Risk-Benefit Analysis. *Food Chem Toxicol* (2020) 146:111788. doi: 10.1016/j.fct.2020.111788.

13. De Cock A, Forio MAE, Croubels S, Dominguez-Granda L, Jacxsens L, Lachat C, et al. Health Risk-Benefit Assessment of the Commercial Red Mangrove Crab: Implications for a Cultural Delicacy. *Sci Total Environ* (2022):160737. Epub 20221208. doi: 10.1016/j.scitotenv.2022.160737.

14. Dellinger MJ, Anguzu R, Pingatore N, Ripley M. Risk-Benefit Modeling to Guide Health Research in Collaboration with Great Lakes Fish Consuming Native American Communities. *J Great Lakes Res* (2020) 46(6):1702-8. doi: 10.1016/j.jglr.2020.08.003.

15. Fan Z, Zou J, Wang Q, Qiu L, Hu G, Song C, et al. Quantitative Benefit and Risk Assessment of Cadmium and Nutrient Levels in Chinese Mitten Crab (Eriocheir Sinensis). *Environmental science and pollution research international* (2021) 28(6):7322-31. doi: 10.1007/s11356-020-10977-9.

16. Fang T, Liang Y, Yang K, Zhao X, Gao N, Li J, et al. Benefit-Risk Assessment of Consuming Fish and Shrimp from a Large Eutrophic Freshwater Lake, China. *Journal of Food Composition and Analysis* (2022) 114:104835. doi: https://doi.org/10.1016/j.jfca.2022.104835.

17. Farzad R, Kuhn DD, Smith SA, O’Keefe SF, Ralston NVC, Neilson AP, et al. Trace Minerals in Tilapia Fillets: Status in the United States Marketplace and Selenium Supplementation Strategy for Improving Consumer’s Health. *PLoS ONE* (2019) 14(6). doi: 10.1371/journal.pone.0217043.

18. Fujimura S, Yoshinaga J. Risk and Benefit of Decreasing Seafood Consumption in Japan&Mdash;Docosahexaenoic Acid, Methylmercury and Infant Iq. *Foods* (2023) 12(8):1674.

19. Girolametti F, Annibaldi A, Carnevali O, Pignalosa P, Illuminati S, Truzzi C. Potential Toxic Elements (Ptes) in Wild and Farmed Atlantic Bluefin Tuna (Thunnus Thynnus) from Mediterranean Sea: Risks and Benefits for Human Consumption. *Food Control* (2021) 125. doi: 10.1016/j.foodcont.2021.108012.

20. Gladyshev MI, Anishchenko OV, Makhutova ON, Kolmakova OV, Trusova MY, Morgun VN, et al. The Benefit-Risk Analysis of Omega-3 Polyunsaturated Fatty Acids and Heavy Metals in Seven Smoked Fish Species from Siberia. *Journal of Food Composition and Analysis* (2020) 90. doi: 10.1016/j.jfca.2020.103489.

21. Grgec AS, Kljakovic-Gaspic Z, Orct T, Ticina V, Sekovanic A, Jurasovic J, et al. Mercury and Selenium in Fish from the Eastern Part of the Adriatic Sea: A Risk-Benefit Assessment in Vulnerable Population Groups. *Chemosphere* (2020) 261. doi: 10.1016/j.chemosphere.2020.127742.

22. Gui Y, Wang Q, Zou J, Chen X, Song C, Chen J. From Pond to Table: Differences in Breeding and Consumption Affect the Balance between Dietary Risk of Residual Cadmium and Uptake Benefits of Nutrients in Chinese Mitten Crab (Eriocheir Sinensis). *Food Chemistry* (2022) 373. doi: 10.1016/j.foodchem.2021.131339.

23. Gui Y, Wang X, Chen X, Wang Q, Yin Y, Song C. Balancing the Health Effect between Risks of Methylmercury and Benefits of Nutrients in Consumption of Chinese Mitten Crab (Eriocheir Sinensis) in China. *Environmental science and pollution research international* (2022) 29(25):38527-34. doi: 10.1007/s11356-022-18822-x.

24. Hasselberg AE, Nostbakken OJ, Aakre I, Madsen L, Atter A, Steiner-Asiedu M, et al. Nutrient and Contaminant Exposure from Smoked European Anchovy (Engraulis Encrasicolus): Implications for Children's Health in Ghana. *Food Control* (2022) 134:11. doi: 10.1016/j.foodcont.2021.108650.

25. Hollander A, De Jonge R, Biesbroek S, Hoekstra J, Zijp MC. Exploring Solutions for Healthy, Safe, and Sustainable Fatty Acids (Epa and Dha) Consumption in the Netherlands. *Sustainability Science* (2019) 14(2):303-13. doi: 10.1007/s11625-018-0607-9.

26. Irigoyen-Arredondo MS, Moreno-Sánchez XG, Escobar-Sánchez O, Soto-Jiménez MF, Marín-Enríquez E, Abitia-Cárdenas LA. Essential (Cu, Zn) and Nonessential (Pb, Cd) Metals in the Muscle of Leopard Groupers (Mycteroperca Rosacea) from a Mining Port in the Gulf of California, Mexico: Human Health Risk Assessment. *Environ Sci Pollut Res* (2022) 29(23):35001-11. doi: 10.1007/s11356-022-18753-7.

27. Jing M, Lin D, Lin J, Li Q, Yan H, Feng X. Mercury, Microcystins and Omega-3 Polyunsaturated Fatty Acids in Farmed Fish in Eutrophic Reservoir: Risk and Benefit Assessment. *Environmental Pollution* (2021) 270:116047. doi: https://doi.org/10.1016/j.envpol.2020.116047.

28. Kendler S, Thornes FW, Jakobsen AN, Lerfall J. Nutritional Profiling and Contaminant Levels of Five Underutilized Fish Species in Norway. *Front Nutr* (2023) 10:1118094. Epub 20230308. doi: 10.3389/fnut.2023.1118094.

29. Kosker AR. Metal and Fatty Acid Levels of Some Commercially Important Marine Species from the Northeastern Mediterranean: Benefits and Health Risk Estimation. *Environ Monit Assess* (2020) 192(6):358. doi: 10.1007/s10661-020-08287-1.

30. Lara A, Galván-Magaña F, Elorriaga-Verplancken FR, Marmolejo-Rodríguez AJ, González-Armas R, Arreola-Mendoza L, et al. Mercury, Selenium and Cadmium in Juvenile Blue (Prionace Glauca) and Smooth Hammerhead (Sphyrna Zygaena) Sharks from the Northwest Mexican Pacific Coast. *Marine Pollution Bulletin* (2022) 175. doi: 10.1016/j.marpolbul.2021.113311.

31. Lazarini TEM, Milani RF, Morgano MA. Selenium, Total Mercury and Methylmercury in Sardine: Study of Molar Ratio and Protective Effect on the Diet. *Journal of environmental science and health Part B, Pesticides, food contaminants, and agricultural wastes* (2019) 54(5):387-93. doi: 10.1080/03601234.2019.1574167.

32. Lin P, Nan FH, Ling MP. Dietary Exposure of the Taiwan Population to Mercury Content in Various Seafood Assessed by a Total Diet Study. *International Journal of Environmental Research and Public Health* (2021) 18(22). doi: 10.3390/ijerph182212227.

33. Lin P, Lee PI, Ling MP. Probabilistic Benefit-Risk Analysis of Fish: Nutritional Benefit Versus Methylmercury-Contaminated Risk. *Marine Pollution Bulletin* (2023) 193. doi: 10.1016/j.marpolbul.2023.115179.

34. Łuczyńska J, Nowosad J, Łuczyński MJ, Kucharczyk D. Evaluation of Chemical Elements, Lipid Profiles, Nutritional Indices and Health Risk Assessment of European Eel (Anguilla Anguilla L.). *International Journal of Environmental Research and Public Health* (2023) 20(3):2257.

35. Łuczyńska J, Łuczyński MJ, Nowosad J, Kowalska-Góralska M, Senze M. Total Mercury and Fatty Acids in Selected Fish Species on the Polish Market: A Risk to Human Health. *International Journal of Environmental Research and Public Health* (2022) 19(16):10092.

36. Maia ML, Almeida A, Soares C, Silva LMS, Delerue-Matos C, Calhau C, et al. Minerals and Fatty Acids Profile of Northwest Portuguese Coast Shrimps. *Journal of Food Composition and Analysis* (2022) 112:104652. doi: https://doi.org/10.1016/j.jfca.2022.104652.

37. Medina-Morales SA, Corro-Espinosa D, Escobar-Sánchez O, Delgado-Alvarez CG, Ruelas-Inzunza J, Frías-Espericueta MG, et al. Mercury (Hg) and Selenium (Se) Content in the Shark Mustelus Henlei (Triakidae) in the Northern Mexican Pacific. *Environ Sci Pollut Res Int* (2020) 27(14):16774-83. doi: 10.1007/s11356-020-08198-1.

38. Melgar MJ, Núñez R, García M. Selenium Intake from Tuna in Galicia (Spain): Health Risk Assessment and Protective Role against Exposure to Mercury and Inorganic Arsenic. *Sci Total Environ* (2019) 694:133716. doi: 10.1016/j.scitotenv.2019.133716.

39. Meng C, Wang K, Xu G. Metals in Ten Commercial Demersal Fish from the East China Sea: Contribution to Aquatic Products Nutrition and Toxic Risk Assessment. *Biological Trace Element Research* (2022) 200(12):5242-50. doi: 10.1007/s12011-021-03087-1.

40. Mesa MD, Gil F, Olmedo P, Gil A. Nutritional Importance of Selected Fresh Fishes, Shrimps and Mollusks to Meet Compliance with Nutritional Guidelines of N-3 Lc-Pufa Intake in Spain. *Nutrients* (2021) 13(2):1-14. doi: 10.3390/nu13020465.

41. Milićević T, Romanić SH, Popović A, Mustać B, Đinović-Stojanović J, Jovanović G, et al. Human Health Risks and Benefits Assessment Based on Ocps, Pcbs, Toxic Elements and Fatty Acids in the Pelagic Fish Species from the Adriatic Sea. *Chemosphere* (2022) 287. doi: 10.1016/j.chemosphere.2021.132068.

42. Mititelu M, Neacșu SM, Oprea E, Dumitrescu DE, Nedelescu M, Drăgănescu D, et al. Black Sea Mussels Qualitative and Quantitative Chemical Analysis: Nutritional Benefits and Possible Risks through Consumption. *Nutrients* (2022) 14(5). doi: 10.3390/nu14050964.

43. Noger-Huet É, Vagner M, Le Grand F, Graziano N, Bideau A, Brault-Favrou M, et al. Risk and Benefit Assessment of Seafood Consumption Harvested from the Pertuis Charentais Region of France. *Environmental Pollution* (2022) 292. doi: 10.1016/j.envpol.2021.118388.

44. Nøstbakken OJ, Rasinger JD, Hannisdal R, Sanden M, Frøyland L, Duinker A, et al. Levels of Omega 3 Fatty Acids, Vitamin D, Dioxins and Dioxin-Like Pcbs in Oily Fish; a New Perspective on the Reporting of Nutrient and Contaminant Data for Risk-Benefit Assessments of Oily Seafood. *Environ Int* (2021) 147:106322. Epub 20201219. doi: 10.1016/j.envint.2020.106322.

45. Okati N, Shahriari Moghadam M, Einollahipeer F. Mercury, Arsenic and Selenium Concentrations in Marine Fish Species from the Oman Sea, Iran, and Health Risk Assessment. *Toxicology and Environmental Health Sciences* (2021) 13(1):25-36. doi: 10.1007/s13530-020-00062-6.

46. Özden Ö, Erkan N, Kaplan M, Karakulak FS. Toxic Metals and Omega-3 Fatty Acids of Bluefin Tuna from Aquaculture: Health Risk and Benefits. *Exposure and Health* (2020) 12(1):9-18. doi: 10.1007/s12403-018-0279-9.

47. Öztürk DK. Element Concentrations of Cultured Fish in the Black Sea: Selenium-Mercury Balance and the Risk Assessments for Consumer Health. *Environmental science and pollution research international* (2022) 29(58):87998-8007. doi: 10.1007/s11356-022-21914-3.

48. Peycheva K, Panayotova V, Stancheva R, Makedonski L, Merdzhanova A, Cicero N, et al. Trace Elements and Omega-3 Fatty Acids of Black Sea (Bulgaria) Bivalve Species Mytilus Galloprovincialis, Chamelea Gallina and Donax Trunculus. Human Health Risk. *Natural Product Research* (2021). doi: 10.1080/14786419.2021.1921770.

49. Peycheva K, Panayotova V, Stancheva R, Makedonski L, Merdzhanova A, Cicero N, et al. Trace Elements and Omega‐3 Fatty Acids of Wild and Farmed Mussels (Mytilus Galloprovincialis) Consumed in Bulgaria: Human Health Risks. *International Journal of Environmental Research and Public Health* (2021) 18(19). doi: 10.3390/ijerph181910023.

50. Peycheva K, Panayotova V, Stancheva R, Makedonski L, Merdzhanova A, Cicero N, et al. Trace Elements and Omega-3 Fatty Acids of Black Sea (Bulgaria) Bivalve Species Mytilus Galloprovincialis, Chamelea Gallina and Donax Trunculus. Human Health Risk. *Natural Product Research* (2022) 36(11):2735-42. doi: 10.1080/14786419.2021.1921770.

51. Plessl C, Gilbert BM, Sigmund MF, Theiner S, Avenant-Oldewage A, Keppler BK, et al. Mercury, Silver, Selenium and Other Trace Elements in Three Cyprinid Fish Species from the Vaal Dam, South Africa, Including Implications for Fish Consumers. *Sci Total Environ* (2019) 659:1158-67. doi: 10.1016/j.scitotenv.2018.12.442.

52. Prato E, Biandolino F, Parlapiano I, Giandomenico S, Denti G, Calò M, et al. Proximate, Fatty Acids and Metals in Edible Marine Bivalves from Italian Market: Beneficial and Risk for Consumers Health. *Science of the Total Environment* (2019) 648:153-63. doi: 10.1016/j.scitotenv.2018.07.382.

53. Harb Rabia S, Luzardo OP, Pozo R, Abbassy M, Zumbado M, Elalfy I, et al. Determination of Heavy Metals from Aloe Vera by- Product in Golden Mullet (Liza Aurata); a Consumer Health Risk Assessment. *Food and Chemical Toxicology* (2022) 169. doi: 10.1016/j.fct.2022.113418.

54. Rajkowska-Myśliwiec M, Pokorska-Niewiada K, Witczak A, Balcerzak M, Ciecholewska-Juśko D. Health Benefits and Risks Associated with Element Uptake from Grilled Fish and Fish Products. *Journal of the science of food and agriculture* (2022) 102(3):957-64. doi: 10.1002/jsfa.11429.

55. Ralston NVC, Kaneko JJ, Raymond LJ. Selenium Health Benefit Values Provide a Reliable Index of Seafood Benefits Vs. Risks. *Journal of Trace Elements in Medicine and Biology* (2019) 55:50-7. doi: 10.1016/j.jtemb.2019.05.009.

56. Ricketts P, Voutchkov M, Chan HM. Risk-Benefit Assessment for Total Mercury, Arsenic, Selenium, and Omega-3 Fatty Acids Exposure from Fish Consumption in Jamaica. *Biol Trace Elem Res* (2020) 197(1):262-70. Epub 20191112. doi: 10.1007/s12011-019-01965-3.

57. Roila R, Branciari R, Ranucci D, Stramenga A, Tavoloni T, Stecconi T, et al. Risk Characterization and Benefit–Risk Assessment of Brominated Flame Retardant in Commercially Exploited Freshwater Fishes and Crayfish of Lake Trasimeno, Italy. *International Journal of Environmental Research and Public Health* (2021) 18(16). doi: 10.3390/ijerph18168763.

58. Sabino MA, Bodin N, Govinden R, Arrisol R, Churlaud C, Pethybridge H, et al. The Role of Tropical Small-Scale Fisheries in Trace Element Delivery for a Small Island Developing State Community, the Seychelles. *Marine Pollution Bulletin* (2022) 181. doi: 10.1016/j.marpolbul.2022.113870.

59. Sardenne F, Bodin N, Medieu A, Antha M, Arrisol R, Le Grand F, et al. Benefit-Risk Associated with the Consumption of Fish Bycatch from Tropical Tuna Fisheries. *Environmental Pollution* (2020) 267. doi: 10.1016/j.envpol.2020.115614.

60. Sepúlveda CH, Sotelo-Gonzalez MI, García-Ulloa M, Góngora-Gómez AM, Espinosa-Alonso LG, Sánchez-Cárdenas R, et al. Mercury and Selenium in Oysters Saccostrea Palmula and Crassostrea Corteziensis from Coastal Lagoons of the Southeastern Gulf of California: Molar Ratio and Risk Assessment on Human Health. *Bulletin of environmental contamination and toxicology* (2023) 111(2):20. doi: 10.1007/s00128-023-03776-8.

61. Simukoko CK, Mwakalapa EB, Bwalya P, Muzandu K, Berg V, Mutoloki S, et al. Assessment of Heavy Metals in Wild and Farmed Tilapia (Oreochromis Niloticus) on Lake Kariba, Zambia: Implications for Human and Fish Health. *Food Additives and Contaminants - Part A Chemistry, Analysis, Control, Exposure and Risk Assessment* (2022) 39(1):74-91. doi: 10.1080/19440049.2021.1975830.

62. Sofoulaki K, Kalantzi I, Machias A, Pergantis SA, Tsapakis M. Metals in Sardine and Anchovy from Greek Coastal Areas: Public Health Risk and Nutritional Benefits Assessment. *Food and Chemical Toxicology* (2019) 123:113-24. doi: 10.1016/j.fct.2018.10.053.

63. Song C, Fang L, Hao G, Xing L, Fan L, Hu G, et al. Assessment of the Benefits of Essential Fatty Acids and Risks Associated with Antimicrobial Residues in Aquatic Products: A Case Study of Chinese Mitten Crab (Eriocheir Sinensis). *Journal of Hazardous Materials* (2023) 451. doi: 10.1016/j.jhazmat.2023.131162.

64. Strandberg U, Bhavsar SP, Arts MT. Interspecific Differences in Omega-3 Pufa and Contaminants Explain the Most Variance in Suggested Great Lakes' Fish Consumption When Risks/Benefits Are Considered Together. *Journal of Great Lakes Research* (2020) 46(3):549-59. doi: 10.1016/j.jglr.2020.03.019.

65. Sun Q, Wang T, Zhan X, Hong S, Lin L, Tan P, et al. Legacy and Novel Perfluoroalkyl Substances in Raw and Cooked Squids: Perspective from Health Risks and Nutrient Benefits. *Environment International* (2023) 177. doi: 10.1016/j.envint.2023.108024.

66. Suyani NK, Rajesh M, Mohan CO, Rajesh KM, Sasikala R, Kishore P. Fatty Acid Profile and Mineral Composition of Red-Toothed Triggerfish (Odonus Niger) Landed in Karnataka, Southeastern Arabian Sea. *Biol Trace Elem Res* (2023). Epub 20230630. doi: 10.1007/s12011-023-03741-w.

67. Thomsen ST, de Boer W, Pires SM, Devleesschauwer B, Fagt S, Andersen R, et al. Health Impact of Substituting Red Meat by Fish: Addressing Variability in Risk-Benefit Assessments. *European Journal of Public Health* (2019) 29:117-+.

68. Tuomisto JT, Asikainen A, Meriläinen P, Haapasaari P. Health Effects of Nutrients and Environmental Pollutants in Baltic Herring and Salmon: A Quantitative Benefit-Risk Assessment. *BMC Public Health* (2020) 20(1):64. doi: 10.1186/s12889-019-8094-1.

69. Uçar Y. Elemental Compositions and Fatty Acid Profiles of Bogue Fish (Boops Boops) from Mediterranean Coast: A Comprehensive Evaluation of the Potential Effects on Human Health. *Biol Trace Elem Res* (2020) 196(1):272-84. doi: 10.1007/s12011-020-02069-z.

70. Ulusoy Ş, Mol S, Karakulak FS, Kahraman AE. Selenium-Mercury Balance in Commercial Fish Species from the Turkish Waters. *Biol Trace Elem Res* (2019) 191(1):207-13. doi: 10.1007/s12011-018-1609-2.

71. Ulusoy Ş, Mol S. Trace Elements in Seabass, Farmed by Turkey, and Health Risks to the Main Consumers: Turkish and Dutch Populations. *Environmental Monitoring and Assessment* (2022) 194(3). doi: 10.1007/s10661-022-09806-y.

72. Varol M, Kaçar E, Sünbül MR, Md Towfiqul Islam AR. Levels of Metals and Elements in Tissues of Fish Species in the Kızılırmak River (Turkey) and Assessment of Health Risks and Nutritional Benefits. *Environmental Research* (2022) 214. doi: 10.1016/j.envres.2022.113791.

73. Varol M, Kaçar E, Sünbül MR, Towfiqul Islam ARM. Species, Tissue and Gender-Related Metal and Element Accumulation in Fish Species in a Large Reservoir (Turkey) and Health Risks and Nutritional Benefits for Consumers. *Environmental Toxicology and Pharmacology* (2022) 94. doi: 10.1016/j.etap.2022.103929.

74. Varol M, Kaçar E. Bioaccumulation of Metals in Various Tissues of Fish Species in Relation to Fish Size and Gender and Health Risk Assessment. *Current Pollution Reports* (2023) 9(3):327-37. doi: 10.1007/s40726-023-00263-w.

75. Wang S, Dong D, Li P, Hua X, Zheng N, Sun S, et al. Mercury Concentration and Fatty Acid Composition in Muscle Tissue of Marine Fish Species Harvested from Liaodong Gulf: An Intelligence Quotient and Coronary Heart Disease Risk Assessment. *Sci Total Environ* (2020) 726:138586. doi: 10.1016/j.scitotenv.2020.138586.

76. Wang P, Chen SW, Chen ZH, Huo WL, Huang R, Huang WX, et al. Benefit-Risk Assessment of Commonly Consumed Fish Species from South China Sea Based on Methyl Mercury and Dha. *Environ Geochem Health* (2019) 41(5):2055-66. Epub 20190226. doi: 10.1007/s10653-019-00254-1.

77. Xie Q, Liu F, Zhang X, Wu Y. Fatty Acids and Organohalogen Contaminants in Seafood from the Pearl River Estuary, China: Risk-Benefit Analyses of Seafood Consumption. *Science of the Total Environment* (2023) 900. doi: 10.1016/j.scitotenv.2023.165725.

78. Yabanli M, Tay S. Selenium and Mercury Balance in Sea Bream Obtained from Different Living Environments in Turkey: A Risk Assessment for the Consumer Health. *Environ Sci Pollut Res Int* (2021). doi: 10.1007/s11356-021-13366-y.

79. Yánez-Jácome GS, Romero-Estévez D, Vélez-Terreros PY, Navarrete H. Total Mercury and Fatty Acids Content in Selected Fish Marketed in Quito – Ecuador. A Benefit-Risk Assessment. *Toxicology Reports* (2023) 10:647-58. doi: 10.1016/j.toxrep.2023.05.009.

80. Yin Y, Chen X, Gui Y, Zou J, Wang Q, Qiu L, et al. Risk and Benefit Assessment of Potential Neurodevelopment Effect Resulting from Consumption of Cultured Largemouth Bass (Micropterus Salmoides) in China. *Environmental science and pollution research international* (2022) 29(59):89788-95. doi: 10.1007/s11356-022-22065-1.

81. Zamora-Arellano NY, Betancourt-Lozano M, Ruelas-Inzunza J, Jara-Marini M, Girón-Pérez MI. Risk and Benefit Analysis of Fish Consumption in Nw Mexico: Mercury, Selenium, and Fatty Acids. *Archives of Environmental Contamination and Toxicology* (2022) 83(1):36-46. doi: 10.1007/s00244-022-00939-2.

82. Zihao F, Qian W, Xi C, Liping Q, Yuting Y, Limin F, et al. Quantitative Benefit and Risk Assessment of Arsenic and Nutrient Levels in Cooked and Raw Chinese Mitten Crab (Eriocheir Sinensis) Using an in Vitro Digestion Model. *Food Chemistry* (2022) 368. doi: 10.1016/j.foodchem.2021.130826.

83. Zou C, Yin D, Wang R. Mercury and Selenium Bioaccumulation in Wild Commercial Fish in the Coastal East China Sea: Selenium Benefits Versus Mercury Risks. *Marine Pollution Bulletin* (2022) 180. doi: 10.1016/j.marpolbul.2022.113754.
